# Metagenomic Shotgun Sequencing Reveals Specific Human Gut Microbiota Associated with Insulin Resistance and Body Fat Distribution in Saudi Women

**DOI:** 10.3390/biom13040640

**Published:** 2023-04-02

**Authors:** Ghadeer S. Aljuraiban, Mohammad A. Alfhili, Madhawi M. Aldhwayan, Esra’a A. Aljazairy, Sara Al-Musharaf

**Affiliations:** 1 Department of Community Health Sciences, College of Applied Medical Sciences, King Saud University, Riyadh 11451, Saudi Arabia; 2 Department of Clinical Laboratory Sciences, College of Applied Medical Sciences, King Saud University, Riyadh 11451, Saudi Arabia

**Keywords:** gut microbiota, insulin resistance, glycemic control, BMI, % body fat, WHR, microbial α-diversity, microbial β-diversity

## Abstract

(1) Background: Gut microbiota dysbiosis may lead to diseases such as insulin resistance and obesity. We aimed to investigate the relationship between insulin resistance, body fat distribution, and gut microbiota composition. (2) Methods: The present study included 92 Saudi women (18–25 years) with obesity (body mass index (BMI) ≥ 30 kg/m^2^, n = 44) and with normal weight (BMI 18.50–24.99 kg/m^2^, n = 48). Body composition indices, biochemical data, and stool samples were collected. The whole-genome shotgun sequencing technique was used to analyze the gut microbiota. Participants were divided into subgroups stratified by the homeostatic model assessment for insulin resistance (HOMA-IR) and other adiposity indices. (3) Results: HOMA-IR was inversely correlated with *Actinobacteria* (r = −0.31, *p* = 0.003), fasting blood glucose was inversely correlated with *Bifidobacterium kashiwanohense* (r = −0.22, *p* = 0.03), and insulin was inversely correlated with *Bifidobacterium adolescentis* (r = −0.22, *p* = 0.04). There were significant differences in α- and β-diversities in those with high HOMA-IR and waist–hip ratio (WHR) compared to low HOMA-IR and WHR (*p* = 0.02, 0.03, respectively). (4) Conclusions: Our findings highlight the relationship between specific gut microbiota at different taxonomic levels and measures of glycemic control in Saudi Arabian women. Future studies are required to determine the role of the identified strains in the development of insulin resistance.

## 1. Introduction

Globally, the prevalence of type 2 diabetes mellitus (T2DM) mellitus is increasing at an alarming rate [1]. This is largely triggered by the rising rates of insulin resistance [2] and the obesity pandemic [3]. As of 2017, T2DM has affected an estimated 462 million people around the world and has led to over 1 million deaths per year, with rates expected to rise [1]. A systematic review and meta-analysis of data from 2000 to 2018 reported a combined prevalence of T2DM of about 15% in Middle Eastern adults [4]. Saudi Arabia is no exception to this crisis, with an estimated national prevalence of 18% among adults aged 20–79 years in 2019 [5]. Regional [6] and nationwide [7,8] studies have also revealed a higher prevalence of T2DM among women than among men. Concurrently, there is a marked increase in the prevalence of obesity globally, with 13% of adults suffering from obesity in 2016 [9]. In the Middle East, in 2016, 50% and 24% of women were estimated to be overweight or obese, respectively [10,11]. The Saudi Arabian Health Interview Survey 2019 also confirmed this finding, revealing a higher prevalence of obesity among women than men (22% vs. 19%, respectively) [12].

The growing prevalence of T2DM and obesity in Saudi Arabia may be partly attributed to the recent population transition from a traditional, healthy lifestyle [13,14] to a more “westernized” one with sedentary behavior and intake of energy-dense diets [15,16]. Furthermore, the typical lifestyle of university students, especially women of child-bearing age, is generally of poor quality [17]. Such conditions could persist later into their lives, putting them at risk of developing diseases [18]. This is particularly concerning since maternal nutritional status is associated with an increased risk of noncommunicable disease in offspring [19]; therefore, steps must be taken to investigate insulin resistance and obesity in the Saudi population to curb and manage the burden of these diseases.

Recently, investigations into the underlying mechanisms responsible for the progression of diseases have suggested that gut microbiota dysbiosis, or altered bacterial composition, is linked to the development of inflammatory diseases and infections [20], insulin resistance [21,22], and obesity [23,24]. Some proposed mechanisms for the relationship with T2DM involve gut microbes that are pro- or anti-inflammatory, reduce gut permeability, influence glucose metabolism by affecting the digestion of sugars, increase fatty acid oxidation, reduce fatty acid synthesis, or affect host physiology by interacting with hepatic, cardiac, and epithelial cell receptors [25,26]. Changes to the gut microbiota can also alter host metabolism to harvest more energy, a key factor in diabetes and obesity development [26].

A recent review noted that *Bifidobacterium, Bacteroides, Faecalibacterium, Akkermansia* and *Roseburia* were commonly found to be negatively associated with T2DM and *Ruminococcus, Fusobacterium*, and *Blautia* were commonly found to be positively associated [25]. In several studies, patients with T2DM showed a decreased abundance of universal butyrate producers and increased opportunistic pathogens [27,28,29]. Furthermore, the *Bacteroidetes/Firmicutes* ratio (B:F) has been proposed as a gut microbial marker for adiposity [30]. Despite this, there are discrepancies between studies regarding the microbial biomarkers and the risk factors underlying these relationships [31,32], attributed mainly to the heterogeneity of groups [32], genetic factors [32], lifestyle habits [33], anthropometric measurements [33], and techniques used to identify the fecal microbiota composition [34]. For instance, some studies use 16S rRNA sequencing while others use shotgun sequencing [35]. Whole-genome shotgun sequencing (WGS) is a metagenomics approach used for taxonomic identification. Metagenomics can be used to detect bacteria, archaea, viruses, and eukaryotes and enables de novo assembly of genomes [35]. Compared to the frequently used 16S rRNA sequencing, WGS was shown to have better microbial resolution, increased accuracy, and was able to identify more microbial species [36].

Although major groups of microorganisms dominate the human gastrointestinal tract, the proportion and composition of species varies considerably between individuals [31]. A recent study investigating the gut microbiota patterns associated with insulin sensitivity in males with overweight or obesity concluded that the gut microbiota varied greatly between cohorts [32]. Consequently, the study found that extrapolating the findings from a single cohort or population into generalized biological relevance may not be appropriate [32]. In fact, researchers applied a model of the gut microbiota of European women with normal, impaired glucose control, or with diabetes to a Chinese cohort and reported that the metagenomic markers for T2DM differed [27].

Thus, population and geography-specific gut microbiota patterns may be necessary when investigating its relationship with health outcomes [27]. Identifying the gut microbiota composition and its association with insulin resistance [32] can help develop strategies that focus on modulating the gut microbiota to promote insulin sensitivity and manage obesity. As such, this study aims to investigate the relationship between insulin resistance, body fat distribution, and gut microbiota in young women in Saudi Arabia using whole-genome shotgun sequencing (WGS).

## 2. Materials and Methods

### 2.1. Sample Population

Methods for this analytical, observational study have been previously published [37]. In summary, between January 2019 and March 2020, female college students aged >18 years, who were either of normal weight (body mass index (BMI); 18.5–24.9 kg/m^2^) or obese (BMI ≥ 30 kg/m^2^) were recruited via emails, the social media network, etc. Subsequently, out of 400 women, 290 were eligible to participate. Vitamins and antibiotic users (n = 88), those with chronic illnesses (n = 20), women who were pregnant (n = 2), those who refused to provide stool samples (n = 193), and participants who had stool samples with DNA concentration below the required level (n = 5), were all excluded. Consequently, 92 female students were included in this study and were invited to visit the study clinic for data collection. Each participant signed a consent form before enrolling in the study. The Institutional Ethics Committee at King Saud University approved this study (KSU-IRB #E-19-3625).

### 2.2. Anthropometric Measurements

Weight was measured twice, in light clothing and without shoes, to the nearest 0.1 kg, immediately after the participants visited the clinic. Height was also measured to the nearest 0.1 cm. BMI was calculated by dividing the weight in kg by the square of height in m^2^. The umbilicus, the lowest rib, and the narrowest waist area were utilized to measure the waist circumference following the World Health Organization standard [9]. At the great trochanter level, the hip circumference was measured using a non-stretchable tape with the legs closely apart. Both readings were recorded to the closest 0.5 cm. If the two measurements differed by >2 cm, a third measurement was made, and the mean of the two most comparative measurements was used. Dividing the mean waist circumference by the mean hip circumference yielded the waist–hip ratio (WHR) which was categorized as follows; low WHR < 0.83 and high WHR ≥ 0.83 [38]. Bioelectrical impedance analysis (BIA) was used to quantify body composition indices (770 BIA; InBody, Seoul, Republic of Korea) [39]. Body fat % (BF%) was considered low or high when BF% ≤ 35% or BF% > 35%, respectively.

### 2.3. Biochemical Measurements

Following an overnight fast of longer than 10 h, blood samples were collected into two 5 mL tubes. Serum was separated and centrifuged within 15 min and sent to the study lab. Fasting blood sugar levels were measured using a biochemical analyzer (Konelab, Espoo, Finland) and insulin levels were measured using a LIAISON XL analyzer (DiaSorin, Saluggia, Italy). The following formula, the homeostatic model assessment of the insulin resistance index (*HOMA-IR*) [40], was used:

$$HOMA-IR=\frac{fasting\;glucose\left(nM\right)\times \mathrm{fasting}\;\mathrm{insulin}\left(µU/L\right)}{22.5}$$

### 2.4. Stool Analysis

Fecal samples were collected aseptically and stored at −80 °C. DNA was extracted from frozen stool and stored at −20 °C for further processing. QIAamp PowerFecal DNA Isolation Kit (Qiagen, Hilden, Germany) was used to extract DNA from 0.25 g aliquots of frozen stool. Following the manufacturer’s protocol, it was then eluted in a 100 μL C6 elution buffer. A NanoDrop spectrophotometer (NanoDrop Technologies, Wilmington, USA) was used to measure purity (260/280 ratio) and concentration of the extracted DNA. The DNA libraries were prepared using the Illumina Nextera XT Library Preparation Kit (Illumina, Inc., San Diego, CA, USA), and samples were sequenced using an Illumina sequencer. For quantitative reading, a Qubit^®^ fluorimeter was used.

### 2.5. Characterization of Microbial Composition

Unrestricted genomic sequencing of all microorganisms present was used to determine the gut microbiota composition; hence, WGS analyses were used [41]. This technique allows for the determination of the gut composition at the major phyla level. Using the CosmosID bioinformatics platform (CosmosID, Inc., Rockville, MD, USA), the unassembled sequencing data were assessed for multi-kingdom microbiome analysis, profiling antibiotic resistance and virulence genes, and quantifying the relative abundance of organisms. With curated genome databases, high-performance data mining, and hundreds of millions of metagenomic sequence reads, this method [42,43,44,45] quickly divides the reads into distinct sequences that produce microbes. Also, the unassembled sequence reads were searched against the curated CosmosID antibiotic resistance and virulence-associated gene databases to identify the community resistome and virulome, referring to the collection of antibiotic resistance and virulence-associated genes in the microbiome, respectively.

### 2.6. Statistical Analyses

IBM SPSS Statistics for Windows (version 24; IBM Corp, Armonk, NY, USA) was used to perform all statistical analyses. The normality of all quantitative variables was tested before the analyses. To determine the impact of insulin resistance, participants were divided into two distinct categories: high and low HOMA-IR, using the median (1.85 mmol/L) as the cut-off point. The independent sample t-test was used to determine the statistical differences between continuous variables, and results are shown as means and standard deviations. Correlation coefficients between gut microbiota and glycemic control were calculated using the Pearson correlation coefficient. A *p*-value of 0.05 was used to demonstrate statistical significance.

In low and high HOMA-IR groups, heatmaps were created using the pheatmap R package [46]. The α-diversity was calculated using the CosmosID taxonomic analysis software (R software Vegan package, version 2.5-6; Oksanen et al., 2019) to measure the gut microbiota richness. The β-diversity was calculated to determine the gut composition from the species-level relative abundance matrices.

To identify the influence of different adiposity indices, the sample was further divided into subgroups stratified by HOMA-IR and low/high adiposity indices (WHR, BF%, and BMI). Then, α- and β-diversity were calculated for all subgroups at the species level. Wilcoxon rank-sum tests were used to calculate the statistical difference between groups for α-diversity by Shannon index, using the ggsignif package for R [47]. For β-diversity, the nonparametric PERMANOVA analysis was used based on the Bray–Curtis distance using Vegan’s function adonis2.

## 3. Results

### 3.1. Descriptive Characteristics

**Figure 1:**
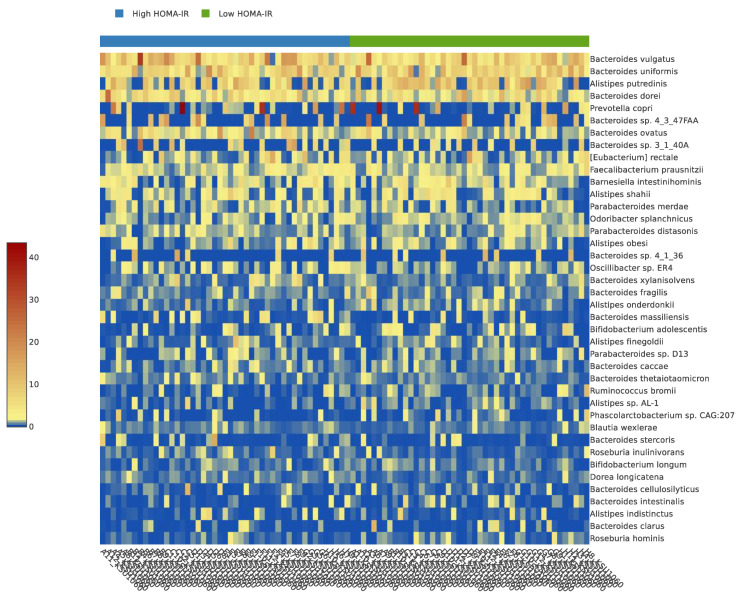
The heatmap analysis of the participants with high and low HOMA-IR.

The current study included a total of 92 participants from Saudi Arabia. Table 1 presents the descriptive traits of participants. Participants known to have high HOMA-IR had higher BMI, BF%, WHR, and WC compared to participants with low HOMA-IR (*p*-value < 0.0001). Fasting blood glucose (*p*-value = 0.04), insulin, total cholesterol (*p*-value = 0.01), LDL (*p*-value = 0.01), HDL (*p*-value = 0.05), and triglycerides (*p*-value < 0.001) were higher in those with high compared to low HOMA-IR. There were no significant differences in the relative abundance of gut microbiota between participants with low and high HOMA-IR (Table 1). The heatmap analysis presents the composition of the gut microbiota of the participants with high and low HOMA-IR (Figure 1). Participants with high HOMA-IR had higher *Bacteroides vulgatus* and *Bacteroides sp. 3_1_40A* and lower *Alistipes putredini* and *Alistipes shahii* compared to those with low HOMA-IR.

### 3.2. Correlation of Microbial Communities with Glycemic Control

HOMA-IR was positively correlated with *Bacteroides_u_s* (r = 0.24, *p* = 0.02) and *Bacteroides thetaiotaomicron* (r = 0.23, *p* = 0.03) and inversely with *Actinobacteria* (r = −0.31, *p* = 0.003), *Bifidobacterium adolescentis* (r = −0.22, *p* = 0.04), and *Bifidobacterium kashiwanohense* (r = −0.21, *p* = 0.05) (Table 2).

Fasting blood glucose was inversely and significantly correlated with *Actinobacteria* (r = −0.20, *p* = 0.05) and *Bifidobacterium kashiwanohense* (r = −0.22, *p* = 0.03) and had an inverse trend with *Proteobacteria* (r = −0.18, *p* = 0.07) and *Bacteroides faecichinchillae* (r = −0.18, *p* = 0.08) (Table 2).

Insulin was positively correlated with *Bacteroides thetaiotaomicron* (r = 0.20, *p* = 0.05) and inversely with *Actinobacteria* (r = −0.28, *p* = 0.01), *Bifidobacterium adolescentis* (r = −0.22, *p* = 0.04), and *Bifidobacterium kashiwanohense* (r = −0.20, *p* = 0.05) (Table 2).

### 3.3. α- and β-Diversity Analysis in Low and High HOMA-IR Groups

**Figure 2:**
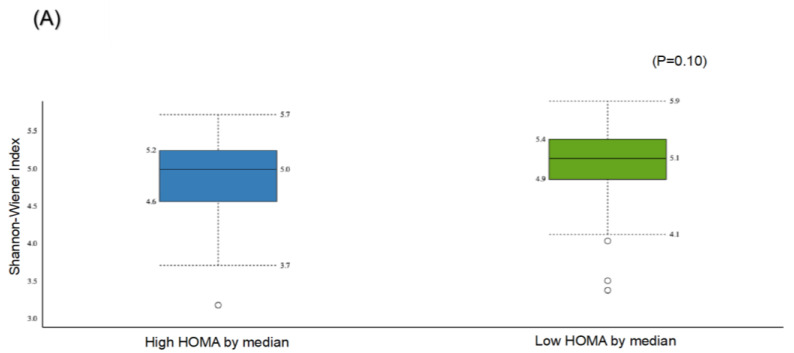

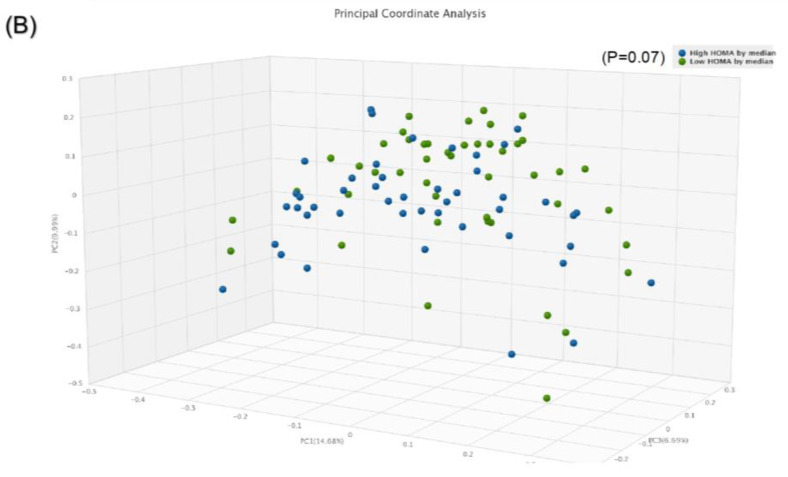
Comparison of bacterial diversities between the gut microbiota of the high HOMA group and the low HOMA group: (**A**) α-diversity, (**B**) β-diversity.

The α-diversity, reflected by the Shannon index, was higher in the group with high HOMA-IR compared to the group with low HOMA-IR (5.1 ± 0.5 vs. 5.0 ± 0.5); however, the difference was not statistically significant (*p*-value = 0.10) (Figure 2A).

The β-diversity using the paired Bray–Curtis similarity index indicated marginal significance between those with high compared to low HOMA-IR (*p* = 0.07) (Figure 2B).

### 3.4. α- and β-Diversity Analysis for Subgroups Stratified by HOMA-IR and Different Adiposity Indices

**Figure 3:**
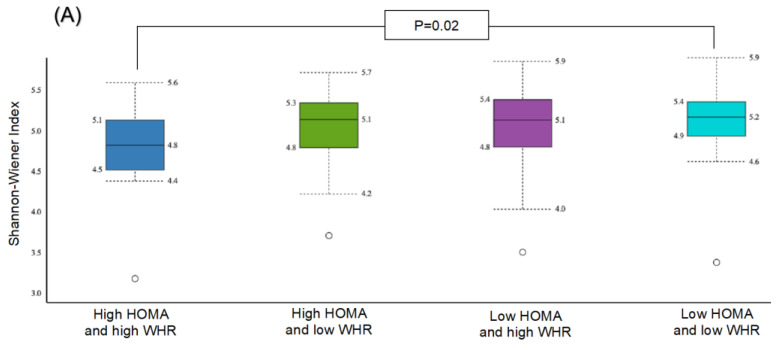

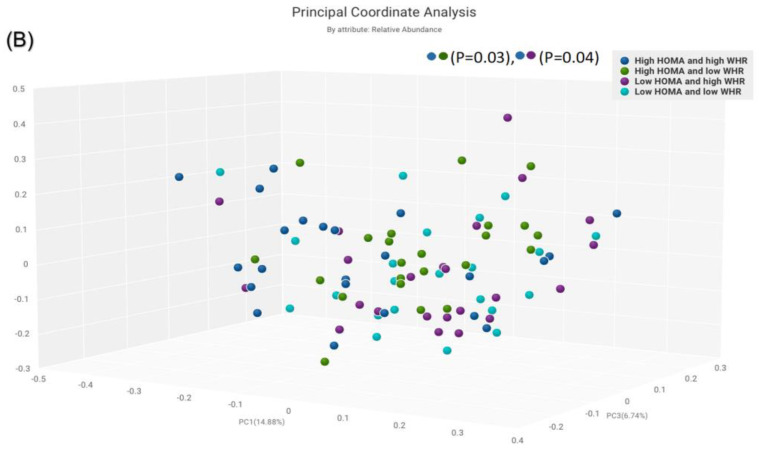
Comparison of bacterial diversities between the gut microbiota of the groups with high HOMA and high WHR, high HOMA and low WHR, low HOMA and high WHR, and low HOMA and low WHR: (**A**) α-diversity, (**B**) β-diversity.

The α-diversity was significant in the subgroup with high HOMA-IR and high WHR compared to the subgroup with low HOMA-IR and low WHR only (*p* = 0.02) (Figure 3A). No statistically significant differences were observed between other subgroups. Similarly, β-diversity was significant for the subgroup with high HOMA-IR and high WHR compared to those with high HOMA-IR and low WHR (*p* = 0.03) and for those with high HOMA-IR and high WHR compared to those with low HOMA-IR and low WHR (*p* = 0.05) (Figure 3B).

**Figure 4:**
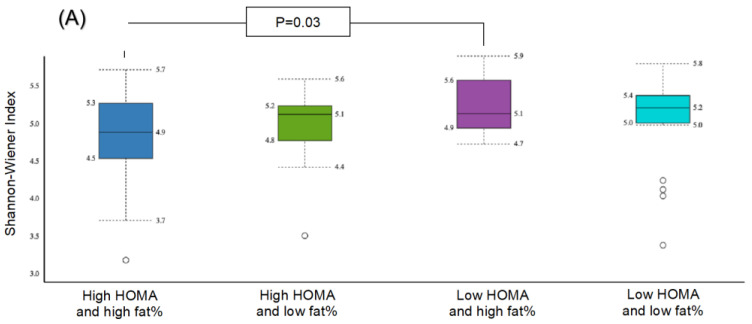

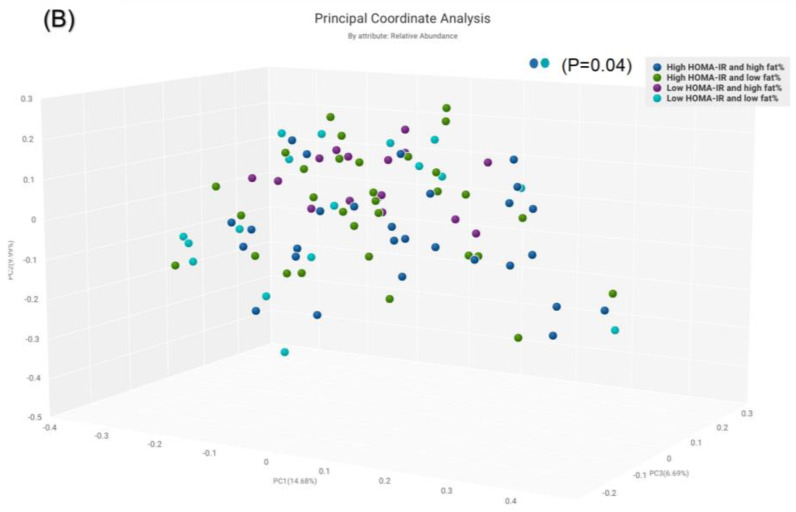
Comparison of bacterial diversities between the gut microbiota of the groups with high HOMA and high fat%, high HOMA and low fat%, low HOMA and high fat% and low HOMA and low fat%: (**A**) α-diversity, (**B**) β-diversity.

For other subgroups, α-diversity was significant between the subgroup with high HOMA-IR and high BF% compared to low HOMA-IR and high BF% (*p* = 0.03) (Figure 4A). The β-diversity was significant for those with high HOMA-IR and high BF% compared to low HOMA-IR and low BF% (*p* = 0.04) (Figure 4B).

**Figure 5:**
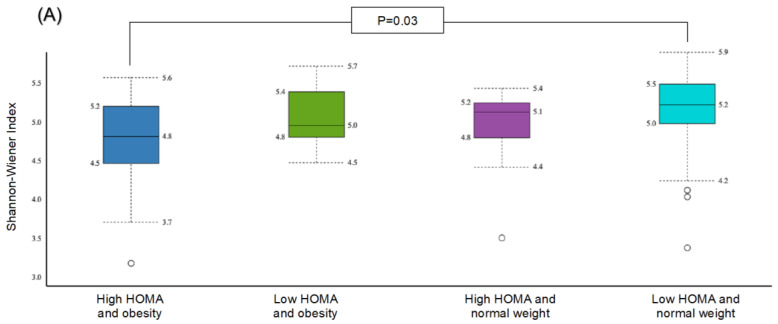

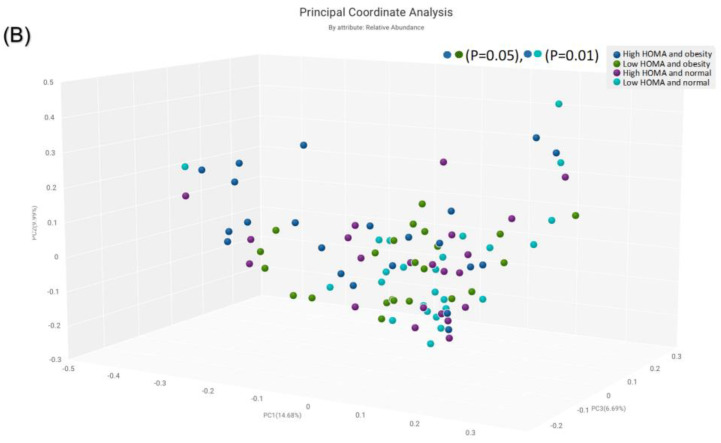
Comparison of bacterial diversities between the gut microbiota of the groups with high HOMA and obesity, low HOMA and obesity, high HOMA and normal weight, and low HOMA and normal weight: (**A**) α-diversity, (**B**) β-diversity.

The α-diversity was significant for the subgroup with obesity and high HOMA-IR in comparison with the subgroup with normal weight and low HOMA-IR (*p* = 0.03) (Figure 5A). The β-diversity was significant for the subgroup with obesity and high HOMA-IR compared to the group with obesity and low HOMA-IR (*p* = 0.05) (Figure 4B), and for the subgroup with obesity and high HOMA-IR in comparison to the subgroup with normal weight and low HOMA-IR (*p* = 0.01) (Figure 5B).

## 4. Discussion

This study aimed to investigate the relationship between insulin resistance, body fat distribution, and gut microbiota in young women in Saudi Arabia using the WGS technique. Results show that specific gut microbiota at different taxonomic levels in Saudi Arabian women are associated with measures of glycemic control, which supports previous hypotheses [48,49]. For instance, some were positively correlated with HOMA-IR *(Bacteroides_u_s, Bacteroides thetaiotaomicron*) and insulin (*Bacteroides thetaiotaomicron*), while others were inversely correlated with HOMA-IR (*Bifidobacterium adolescentis, Bifidobacterium kashiwanohense*), fasting blood glucose (*Actinobacteri, Bifidobacterium kashiwanohense*), and insulin (*Actinobacteria, Bifidobacterium adolescentis, Bifidobacterium kashiwanohense*). Although this work investigated markers of diabetes rather than diabetes itself, the relationships found supporting studies showing that gut microflora differs between individuals with and without T2DM [50]. One study looked at this relationship specifically among patients in Saudi Arabia and found that *Firmicutes* levels were upregulated, and the *Firmicutes/Bacteroidetes* ratio was elevated among those with T2DM compared to those without [51]. Further, the study revealed that both men and women with T2DM had a higher abundance of *prevotellaceae* compared to those without [51], in contrast to earlier findings demonstrating that gut microbiota composition varies between genders [52,53]. In comparison to other populations, studies conducted on Chinese and Egyptian populations showed that the composition of gut microbiota is, in fact, associated with T2DM [54,55]. Some evidence showed that gut microbiota is influenced by geographic location and ethnicity [56,57]. For example, a study by He et al. revealed that the composition of the gut microbiota and its association with metabolic disease were strongly dependent on geography [56]. These data collectively suggest the possible role of gut microbiota in T2DM development.

The results can also be compared to prior work that looked at more specific types of bacteria. For instance, when combined with *Methanobrevibacter smithii* in germ-free mice, *Bacteroides thetaiotaomicron* increased short-chain fatty acid production [58], which has been demonstrated to lead to increased glycemic control [59]. Earlier work has also shown that the *Actinobacteria* phyla, *Bifidobacteria*, was inversely associated with insulin sensitivity [60]. Specifically, in agreement with the present study’s results, *Bifidobacterium adolescentis* has been associated with improved insulin sensitivity in a supplementation study on rats [61]. Further, in a more recent study, *Bifidobacterium adolescentis* was shown to help regulate blood glucose levels by increasing the amount of short-chain fatty acid-producing flora, alleviating inflammation, and restoring gut microbiota homeostasis in mice [62]. In a randomized, double-blind, cross-over trial investigating the effect of probiotic yogurt on metabolic and inflammatory biomarkers compared to acidified milk [63], *Bifidobacterium kashiwanohense* was less abundant after 2 weeks of the intervention with probiotic yogurt intake (*p* < 0.01). Interestingly, in our current work we found that *Bifidobacterium kashiwanohense* was inversely and significantly correlated with HOMA-IR, fasting blood glucose, and insulin. This finding highlights that *Bifidobacterium kashiwanohense* may be a potential biomarker of T2DM. Nevertheless, the role of *Bifidobacterium kashiwanohense* in T2DM development has not been fully identified in the literature, and future investigations are warranted.

When examined independently, there was no significant difference in α- and β-diversity between the low and high HOMA-IR groups. However, α-diversity trended higher in the high HOMA-IR group. The previous study conducted in Saudi Arabia of individuals with and without T2DM also found a trend towards increased diversity among participants with T2DM and higher glucose levels [51], a finding similar to other work from the Middle East [64] but different from results from Western populations. For instance, a population-based study that used two European cohorts found that lower gut microbiota diversity was associated with higher levels of HOMA-IR irrespective of BMI [65]. Discrepancies in the findings between the present study and those previously conducted on different cohorts may be attributable to the differing microbial biomarkers between groups that could result from genetics, lifestyle, sex, age, geography-specific microbiota patterns, or measurement techniques [27,32,33,34,66,67]. Thus, it is imperative to conduct locally representative studies to better understand the relationship between gut microbiota and health measures.

In the present work, adding different adiposity indices to analyses of HOMA-IR levels and diversity allowed for the emergence of significant findings. The nature of these findings appears to depend on the type of diversity and the type of adiposity index examined. For those with a high BF%, the α-diversity differed between those with low and high HOMA-IR. While for some groups, it appears the combination of an adverse HOMA-IR (high) and an adverse adiposity index led to significant differences (i.e., high WHR and obesity for α-diversity and high BF% for β-diversity). For other combinations, β-diversity differed within a HOMA-IR group primarily due to the stratification by a measure of adiposity (i.e., low vs. high WHR for high HOMA-IR and obesity vs. normal weight for low HOMA-IR). These findings suggest the importance of considering adiposity indices in these analyses. A previous study on this same cohort was the first to examine several obesity markers (i.e., WHR, BF%, and obesity) as they relate to gut microbiota among young women in the Middle Eastern region [37]. That study found α- and β-diversity was significantly associated with WHR, and β-diversity was significantly associated with BF% [37]. The results from that work highlight the vital relationship between adiposity measures and microbiota diversity in this cohort and help explain the significance that emerged with the addition of adiposity indices in the present work.

Identifying the association between gut microbiota and insulin resistance can help develop strategies to promote insulin sensitivity and manage obesity. For example, gut microbiota signatures can be used to identify individuals at higher risk for diabetes and act as a target for microbial alterations to improve insulin sensitivity. In fact, recent work has demonstrated promising results for treating diabetes with fecal microbiota transplantation in mice [68]. Such efforts are particularly essential given the growing prevalence of T2DM in Saudi Arabia.

A strength of this work is that the study used the high-standard WGS method to determine gut microbiota at the species level, leading to higher sensitivity and resolution than other techniques. Repeated measurement tools were used to increase the accuracy of data collection methods. Furthermore, a combination of tools to measure microbial diversity (richness and variation) was used. On the other hand, this observational study does not allow for conclusions about causation, and there is a possibility of measurement error. Furthermore, the generalizability of these findings is additionally limited since only women participants in a single setting were included. However, selecting a specific cohort addresses the inability to generalize results from other cohorts to this population.

In conclusion, the present study was the first to examine markers of glycemic control, obesity, and gut microbiota among young women in Saudi Arabia. The results support others conducted in Saudi Arabia and Middle Eastern countries highlighting the benefit of using geography-specific cohorts. Lastly, the association between glycemic measures and microbial diversity was further enhanced by the inclusion of measures of adiposity. Our findings highlight the need for further clinical studies to investigate T2DM treatment through manipulation of gut microbiota approaches, such as the use of short-chain fatty acids, probiotics, prebiotics in the diet, fecal bacteria transplantation, or antibiotics. Such approaches will allow meaningful progress in the quest to understand the role of gut microbiota in the development of T2DM and will be an innovative step in the prevention and treatment of T2DM.

## Figures and Tables

**Table 1:** Characteristics of participants stratified by HOMA-IR, n = 92 *.

	Low HOMA-IR (<1.85 mmol/L)	High HOMA-IR (≥1.85mmol/L)	*p* Value
*n*	49	43	
Age (years)			0.14
Body composition indices			
BMI (kg/m^2^)	24.2 (5.4)	33.6 (7.5)	<0.0001
Skeletal muscle mass (kg)	28 (9.3)	28.8 (7.6)	0.62
WHR (ratio)	0.7 (0.1)	0.8 (0.1)	<0.0001
Waist (cm)	70.2 (10.6)	92.4 (16)	<0.0001
Body fat (%)	37.9 (8.0)	47.9 (7.8)	<0.0001
Biochemical data			
HOMA-IR (mmol/L)	1.1 (0.4)	3.6 (1.2)	<0.0001
Insulin (µIU/mL)	6.5 (2.9)	16.5 (5.3)	<0.0001
Fasting blood glucose (mmol/L)	4.2 (0.9)	4.9 (0.5)	<0.0001
Total cholesterol (mmol/L)	3.7 (1.7)	4.4 (1.1)	<0.0001
HDL cholesterol (mmol/L)	1.0 (0.4)	1.0 (0.3)	0.56
LDL cholesterol (mmol/L)	2.6 (1.5)	3.2 (1.1)	<0.0001
Triglyceride (mmol/L)	0.6 (0.3)	0.9 (0.4)	<0.0001
Gut Microbiota Composition			
*Actinobacteria*	0.0435 (0.0336)	0.0334 (0.0311)	0.14
*Akkermansia muciniphila*	0.00667 (0.0146)	0.00284 (0.00579)	0.11
*Bacteroides (unidentified phylum)*	0.000968 (0.00169)	0.00054 (0.000793)	0.13
*Bacteroides (unidentified species)*	0.00314 (0.00477)	0.00542 (0.00975)	0.15
*Bacteroides faecichinchillae*	0.000255 (0.000337)	0.000253 (0.000252)	0.45
*Bacteroides thetaiotaomicron*	0.00729 (0.00556)	0.00988 (0.00947)	0.11
*Bacteroides uniformis*	0.0705 (0.0438)	0.0676 (0.0424)	0.75
*Bacteroidetes*	0.6901 (0.1359)	0.7202 (0.1131)	0.26
*Bifidobacterium adolescentis*	0.011 (0.0153)	0.0071 (0.011)	0.17
*Bifidobacterium kashiwanohense*	0.0012 (0.00223)	0.000751 (0.00161)	0.27
*Bifidobacterium longum*	0.00687 (0.00571)	0.00663 (0.00787)	0.86
*Bifidobacterium merycicum*	0.000234 (0.000235)	0.00012 (0.000524)	0.14
*Bifidobacterium pseudocatenulatum*	0.003 (0.00574)	0.00256 (0.00536)	0.71
*Blautia wexlerae*	0.0065 (0.00554)	0.00812 (0.00561)	0.17
*Clostridium Bolteae*	0.000708 (0.00125)	0.00091 (0.00216)	0.58
*Clostridium difficile*	0.000244 (0.000248)	0.000178 (0.000638)	0.2
*F:B ratio*	0.419 (0.4058)	0.3507 (0.2406)	0.34
*Faecalibacterium Prausnitzii*	0.0217 (0.0137)	0.0207 (0.0114)	0.73
*Firmicutes*	0.2422 (0.1121)	0.2277 (0.0954)	0.51
*Flavonifractor plautii*	0.00099 (0.00139)	0.00118 (0.0015)	0.53
*Fusobacteria*	0 (0)	0.00021 (0.0013)	0.26
*Lactobacillus acidophilus*	0.000265 (0.000266)	0.000266 (0.000267)	0.7
*Proteobacteria*	0.0149 (0.00977)	0.0146 (0.0144)	0.92
*Verrucomicrobia*	0.00648 (0.0146)	0.00282 (0.0058)	0.13

* Data presented as mean (SD) for normal variables and median. Body mass index (BMI), *Firmicutes/Bacteroidetes* (F:B), high-density lipoprotein (HDL), homeostatic model assessment of the insulin resistance index (HOMA-IR), low-density lipoprotein (LDL), waist-to-hip ratio (WHR).

**Table 2:** Correlation between gut microbiota and glycemic control *.

	HOMA-IR (mmol/L)		Fasting Blood Glucose (mmol/L)		Insulin (µIU/mL)	
	R	*p*-Value	R	*p*-Value	R	*p*-Value
*Actinobacteria*	−0.31	0.003	−0.20	0.05	−0.28	0.01
*Akkermansia muciniphila*	−0.15	0.15	−0.07	0.52	−0.17	0.11
*Bacteroides (unidentified phylum)*	−0.04	0.71	0.07	0.51	−0.05	0.66
*Bacteroides (unidentified species)*	0.24	0.02	0.14	0.19	0.18	0.08
*Bacteroides faecichinchillae*	−0.04	0.71	−0.18	0.09	−0.01	0.98
*Bacteroides thetaiotaomicron*	0.23	0.03	0.16	0.13	0.20	0.05
*Bacteroides uniformis*	−0.04	0.70	−0.14	0.19	0.03	0.75
*Bacteroidetes*	0.18	0.08	0.08	0.47	0.17	0.11
*Bifidobacterium adolescentis*	−0.22	0.04	−0.13	0.21	−0.22	0.04
*Bifidobacterium kashiwanohense*	−0.21	0.05	−0.22	0.03	−0.20	0.05
*Bifidobacterium longum*	−0.17	0.10	−0.16	0.12	−0.12	0.24
*Bifidobacterium merycicum*	−0.02	0.89	0.01	0.92	−0.02	0.85
*Bifidobacterium pseudocatenulatum*	−0.03	0.76	0.01	0.92	−0.06	0.57
*Blautia wexlerae*	0.04	0.68	−0.08	0.44	0.11	0.32
*Clostridium Bolteae*	0.07	0.49	0.12	0.24	0.07	0.48
*Clostridium difficile*	−0.01	0.96	−0.02	0.86	0.01	0.97
*F:B ratio*	−0.10	0.32	−0.01	0.90	−0.10	0.33
*Faecalibacterium Prausnitzii*	−0.04	0.71	0.04	0.73	−0.06	0.54
*Firmicutes*	−0.09	0.40	−0.01	0.98	−0.08	0.47
*Flavonifractor plautii*	0.06	0.55	0.12	0.25	0.06	0.60
*Fusobacteria*	0.16	0.14	0.01	0.93	0.18	0.08
*Lactobacillus acidophilus*	0.01	0.92	0.02	0.84	0.04	0.73
*Proteobacteria*	−0.15	0.15	−0.18	0.08	−0.13	0.23
*Verrucomicrobia*	−0.14	0.17	−0.07	0.51	−0.16	0.12

* Firmicutes/Bacteroidetes (F:B).

## Data Availability

The datasets generated for this study can be found in the Figshare repository: 10.6084/m9.figshare.20106176.
